# The complete mitochondrial genome information of *Phoxinus phoxinus* (Cypriniformes: Cyprinidae) on the Korean Peninsula and the phylogenetic implication

**DOI:** 10.1080/23802359.2019.1687021

**Published:** 2019-11-06

**Authors:** Yoon Jeong Lee, Sun Ho Cha, Junghwa An, Ho Young Suk

**Affiliations:** a Department of Life Sciences, Yeungnam University, Gyeongsan, South Korea;; b GenoTech Corporation, Daejeon, South Korea;; c National Institute of Biological Resources, Environmental Research Complex, Incheon, South Korea

**Keywords:** *Phoxinus phoxinus*, Leuciscinae, mitogenome, Korean Peninsula, phylogeny

## Abstract

*Phoxinus phoxinus* is a small Leuciscinae species predominantly found in cool and well-oxygenated streams throughout a wide area encompassing Europe, Siberia and East Asia. It is believed that the populations in Korea hold important clues to how the species has been distributed south along the Eurasian continent to the Korean Peninsula. We characterized the complete mitochondrial genomes of two individual fin-clip samples collected from the two Korean river systems. The whole sequences were 17,665 and 18,220 bp, respectively, and included 13 protein-coding genes, 2 ribosomal RNA genes and 22 transfer RNA genes. The genome size difference was due to the considerably different sizes of the control region. The overall genome structures were identical to those observed in other Leuciscinae species.

*Phoxinus phoxinus* is a small cyprinid (Leuciscinae) species predominantly found in cool and well-oxygenated streams throughout a wide area encompassing Europe, Siberia and East Asia (Sakai et al. 2006; Collin and Fumagalli 2011). South Korea is the southern limit of its distribution. This species is observed in two different river systems, the Han River, which flows west into the Yellow Sea, and the Samcheogoship River, which drains into the east coast, in South Korea (Song and Son 2002). These two river systems are presumed to historically originate in different regions, the Yellow River in China (Han) and the Amur River in Russia (Samcheogoship; Jeon and Suk 2014; Bae and Suk 2015). The genetic structure of this species can thus provide important clues for elucidating the biogeographical dispersal pathways to the Korean Peninsula along the Eurasian continent. Here, we characterized the complete mitochondrial genomes of two individual fin-clip samples representatively collected from the Han (37°22′40.0″N 128°38′58.5″E) and Samcheogoship River (37°25′19.0″N 129°06′26.1″E) under the official permission of the Korean Ministry of Environment. The remaining DNA samples were stored as vouchered specimens (NIBRGR0000599057: Han; NIBRGR0000599087: Samcheogoship) in Korean National Institute of Biological Resources.

The genomic structure was initially determined using MITOS web server (Bernt et al. 2013) and defined after manually comparing with the mitogenomes of other related species (Imoto et al. 2013). The complete mitogenome sequences were deposited at NCBI GenBank under the accession numbers MK227442 (Han) and MK227443 (Samcheogoship), respectively. The whole sequences were 17,665 (Han; G + C content: 43.7%) and 18,220 (Samcheogoship; G + C content: 43.7%) bp, respectively, and included 13 protein-coding genes, 2 ribosomal RNA genes and 22 transfer RNA genes. The genome size difference was due to the considerably different sizes of the control region (1989 *vs.* 2544 bp). Every protein-coding gene contained ATG start codon with two exceptions in COX1 and ND3 starting with GTG in both mitogenomes. The stop codon was varied by locus; the five loci, ND1, COX1, APT6, ND4L and ND6, were terminated by TAA, the two loci, ATP8 and ND5, by TAG and ND6 by AGA. Incomplete stop codon was detected at COX2 (CTA), COX3 (CCT), ND2 (CCT or TCT), ND3 (AAT), ND4 (CTA) and Cyt *b* (ATA). L-strand was detected in eight tRNA genes and ND6 in both mitogenomes. Based on 13 protein-coding genes, we examined the evolutionary relationship of Korean *P. phoxinus* with other related species (Imoto et al. 2013; Figure 1). The two Korean *P. phoxinus* were the closest to each other and were placed to be the sister to *P. p. tumensis* (KC992395; Xu et al. 2014; Figure 1). Mongolian *P. phoxinus* (M; Imoto et al. 2013) and *P. p. ujmonensis* (NC023802; Xie et al. 2016) formed a monophyletic group, being placed as the sister to Korean *P. phoxinus* and *P. p. tumensis* group (Figure 1). European *P. phoxinus* (I; Imoto et al. 2013) was placed at the basal in the clade (Figure 1).

**Figure 1.**
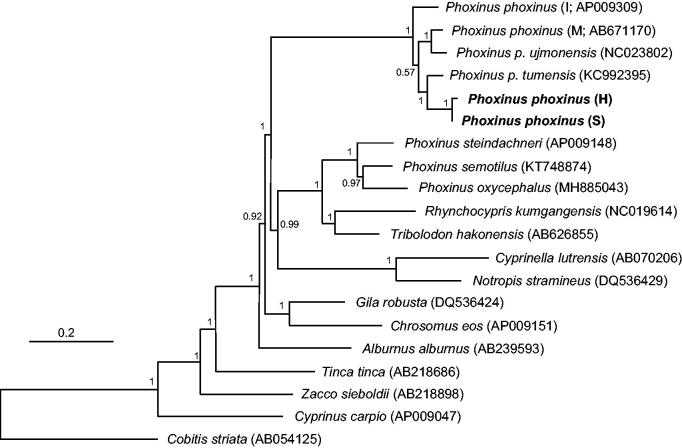
Bayesian inference for the phylogenetic placement of Korean *Phoxinus phoxinus* (H: Han; S: Samcheogoship; bolded) among Leuciscinae species reconstructed by MrBayes 3.2 (Ronquist et al. 2012) using 13 protein-coding mitochondrial genes. *Cobitis striata* (Cobitidae) was used as an outgroup. Each parentheses next to the species name indicates the NCBI GenBank accession number. *GTR + I + G* was selected as the best-fit substitution model by jModeltest 2.1.4 (Darriba et al. 2012) under Akaike information criterion (Akaike 1974), and two parallel runs were performed for two million Markov Chain Monte Carlo (MCMC) generations with sampling every 1,000 steps. Posterior probabilities were indicated on the nodes.
